# Implementation of holistic nursing interventions based on fast track surgery concept in patients with lower extremity arterial occlusive disease

**DOI:** 10.1097/MD.0000000000036485

**Published:** 2023-12-08

**Authors:** Jie Zhang, Guojun Zeng, Lin Zhang, Jing Huang, Xiaoyan Liu

**Affiliations:** a Department of Vascular Surgery, West China Hospital, Sichuan University, Chengdu, Sichuan Province 610041, China.

**Keywords:** fast track surgery, holistic nursing interventions, lower extremity arterial occlusive disease, patient adherence, sleep quality

## Abstract

Lower Extremity Arterial Occlusive Disease (LEAOD) is a prevalent condition affecting many patients worldwide, which requires careful management and patient cooperation. This study aimed to evaluate the effectiveness of holistic nursing interventions based on the Fast Track Surgery (FTS) concept in patients with LEAOD. A retrospective analysis of 92 LEAOD patients, randomized into control and experimental groups, was performed. Conventional rehabilitation nursing interventions were applied to the control group, while the experimental group received holistic rehabilitation nursing interventions based on the FTS concept. Patient adherence was assessed before and after the intervention using a hospital survey, and sleep quality was evaluated using the Pittsburgh Sleep Quality Index on days 3, 7, and 15 post-interventions. Post-intervention, the experimental group exhibited significantly improved adherence to balanced diet, regular exercise, timely medication, and regular review visits compared to the control group (*P* < .05). Further, Pittsburgh Sleep Quality Index scores indicated significantly better sleep quality over time in the experimental group than in the control group (*P* < .05). The implementation of holistic nursing interventions based on the FTS concept significantly improved patient adherence and sleep quality in LEAOD patients. These findings highlight the potential benefits of integrating such interventions in the management of LEAOD patients, potentially enhancing postoperative recovery and overall health outcomes.

## 1. Introduction

Lower extremity arterial occlusive disease (LEAOD) represents a significant health challenge, arising as a consequence of atherosclerosis, which leads to a narrowing or blockage of the arteries in the lower extremities.^[1,2]^ This results in sustained ischemia and hypoxia-induced necrosis within localized regions of the lower limbs. The manifestation of LEAOD is often marked by an array of distressing symptoms including, but not limited to, lower limb numbness, weakness, rest pain, and intermittent claudication. These symptoms can considerably impair the patient’s mobility and independence, thereby adversely affecting their overall quality of life.^[3,4]^ Surgical interventions have proven to be an effective cornerstone in the therapeutic arsenal against LEAOD. Despite the success of surgical treatment in directly alleviating the disease’s clinical symptoms, it is recognized that the postoperative period represents a critical juncture for recovery and rehabilitation. However, postoperative patient compliance often falls short due to factors such as a lack of comprehensive knowledge about the disease, the surgical procedure, and the subsequent recovery process. Moreover, the pain and discomfort associated with the surgical wound further exacerbate the challenge of adherence to postoperative care guidelines.^[5,6]^

In view of these postoperative challenges, the emphasis on comprehensive, high-quality nursing care is paramount to optimize patient recovery. The contemporary paradigm of holistic nursing intervention has emerged as a significant focus area in this regard, particularly when combined with the principles of Fast Track Surgery (FTS). FTS, also known as Enhanced Recovery After Surgery, embodies a multi-disciplinary, multi-modal approach that aims to minimize surgical stress, optimize perioperative care, and thereby expedite postoperative recovery.^[7,8]^ With respect to LEAOD, this amalgamation of holistic nursing care and FTS principles offers a compelling prospect.^[9]^ The multi-dimensional approach addresses the physical, psychological, social, and spiritual needs of the patient, enhancing their overall well-being. Furthermore, by bolstering patient knowledge about the disease and its management, this approach seeks to improve patient compliance and engagement in the postoperative recovery process. By mitigating the adverse impact of wound pain through effective strategies, the holistic can enhance functional recovery and sleep quality.^[10,11]^ Despite the potential benefits of this integrative approach, several areas need to be addressed to ensure its effective implementation. This includes the need for further research on optimizing nursing care strategies, training for healthcare professionals, patient education, and addressing potential barriers to implementation.

This paper aims to provide a comprehensive review of the current understanding of LEAOD, and the challenges associated with its management. We propose a structured approach for implementing holistic nursing interventions based on the FTS concept in the care of patients with LEAOD. We also discuss the potential barriers to implementation and propose strategies to overcome these. The objective is to contribute to an improved standard of care for patients with LEAOD, ultimately enhancing patient satisfaction, compliance, functional recovery, and quality of life.

## 2. Methods

### 2.1. Study design and population

**Figure 1.**
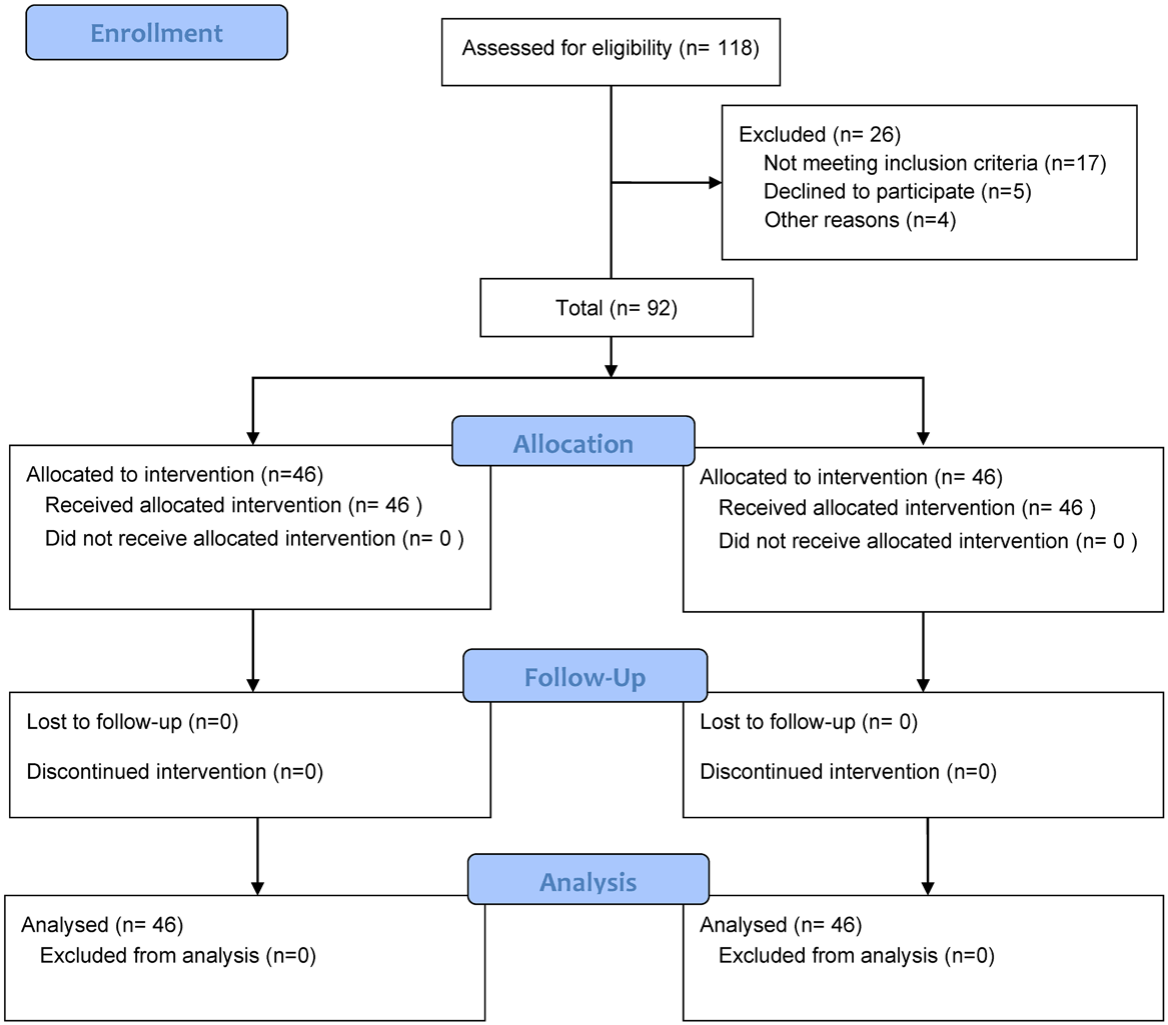
Flow diagram of the study design and population.

This research was approved by the Medical Ethics Committee. A retrospective analysis was performed on 92 patients with LEAOD admitted to our vascular surgery department from November 2020 to December 2022. Patients were divided into a control group and an experimental group, each consisting of 46 individuals (Fig. 1).

Inclusion criteria encompassed those who (1) met the diagnostic criteria for LEAOD through angiography, magnetic resonance angiography, color Doppler ultrasound, and serum lipid laboratory examination; (2) had Fontaine stage I to III disease; and (3) had complete clinical data and signed an informed consent form. Exclusion criteria included: (1) a family history of severe mental illness within the last 3 generations; (2) severe lower extremity infection leading to sepsis or septicemia; (3) concomitant severe heart valve disease, acute coronary syndrome, severe liver and kidney dysfunction or frostbite injury syndrome; and (4) severe necrosis, edema, or ulceration of the lower limbs. No statistically significant differences in age, gender, disease course, or Fontaine staging were found between the 2 groups (*P* > .05), thus demonstrating comparability between the groups (Table 1).

**Table 1:** Comparison of baseline characteristics between control and experimental groups.

Group (n)	Age (years, mean ± SD)	Gender (male/female, n)	Disease duration (years, mean ± SD)	Fontaine stage (I/II/III, n)
Experimental (46)	68.80 ± 3.54	24/22	3.48 ± 0.34	22/19/5
Control (46)	68.30 ± 3.50	23/23	3.44 ± 0.44	19/19/8
t/χ2-value	0.129	0.051	0.220	0.265
*P*-value	.898	.870	.830	.890

### 2.2. Nursing interventions

#### 2.2.1. Control group.

Conventional rehabilitation nursing interventions were implemented. The department’s head nurse supervised these interventions, which were conducted by the responsible nursing staff. Patients were informed about the disease-related knowledge and were guided through preoperative examinations, intraoperative coordination, and postoperative timed and measured rehabilitation training.

#### 2.2.2. Experimental group.

A holistic rehabilitation nursing intervention based on the FTS concept was implemented. A rehabilitation nursing team was established, including a senior resident physician specialized in vascular surgery, a head nurse, 4 specialist responsible nurses, and a rehabilitation therapist. The nursing staff utilized academic websites like PubMed to retrieve professional literature related to “lower extremity arterial occlusive disease,” “rehabilitation exercise,” and “sleep quality.” Information gathered from the literature was presented in the form of videos and images, and one-on-one explanations were given to newly admitted patients at their bedside. The nursing team also encouraged patients to voice their concerns and anxieties, summarized these problems, and deliberated within the group to formulate improvement measures. During surgery, nursing staff assisted the surgeon to maximize the exposure of the surgical field. They placed soft pads at the bony prominences such as the ankle and knee joints to avoid the great saphenous vein. Nonoperative areas were wrapped in cotton pads or heated electric blankets maintained at 40 to 42 °C. The wound cleansing solution used was warm saline at 38 to 40 °C, with vital signs monitored every 15 minutes. Postoperatively, patients were guided to perform early ankle pump exercises on the bed for 10 to 15 minutes within 6 hours after the surgery. After each exercise session, patients rested for 30 to 35 minutes. This was followed by leg raising, toe dorsiflexion, and leg curl resistance training using a yellow TheraBand elastic band. Each exercise was performed in sets of 15 repetitions, 2 to 3 sets in total. Patients were encouraged to engage in early ambulation in their room or the hallway, starting with 15 minutes and gradually transitioning to 25 to 30 minutes, 2 to 3 times a day for 10 to 15 days. Ice packs were applied to the area around the wound for 1 to 20 minutes, with a 2-hour interval between each application, continuously for 24 hours. Patients were guided to perform mindfulness relaxation techniques and positive association or reminiscence for 20 to 25 minutes twice a day, morning and evening.

### 2.3. Outcome measures and evaluation criteria

Patient adherence was assessed before and after the intervention using a self-made hospital survey that evaluated dimensions such as balanced diet, persistent exercise, regular medication, and regular reviews. Each dimension had a maximum score of 25, with the total score positively correlated with adherence behavior (Cronbach alpha = 0.82).^[6]^ Sleep quality was assessed on days 3, 7, and 15 post-intervention using the Pittsburgh Sleep Quality Index (PSQI), which evaluates dimensions such as sleep disturbances, sleep duration, and sleep efficiency. The total score was negatively correlated with sleep quality (Cronbach alpha = 0.772).^[7]^

### 2.4. Statistical analysis

Data obtained was analyzed using SPSS 26.0. Measurement data was expressed as mean ± standard deviation (x ± s), and comparisons were made using the *t*-test, with repeated measures analysis of variance used for multiple time-point comparisons. Count data was represented as case number and percentage, and comparisons were made using the χ2 test. A *P*-value of <.05 was considered statistically significant.

### 2.5. Treatment satisfaction assessment

To evaluate the level of patient satisfaction with the implemented nursing interventions, a treatment satisfaction survey was utilized post-intervention. This tool was designed to measure patients’ satisfaction in 3 distinct categories: (1) satisfied: This category captured the patients who felt that the nursing interventions met or exceeded their expectations and that their health outcomes had significantly improved; (2) basic satisfaction: patients in this category believed that the nursing interventions had somewhat met their expectations, providing them with a moderate improvement in health outcomes. (3) Dissatisfied: patients who felt that the nursing interventions did not meet their expectations and had little to no improvement in health outcomes were grouped here.

The overall satisfaction rate was derived by summing the patients in the “Satisfied” and “Basic Satisfaction” categories. This measure was used to gain a holistic view of the overall contentment of the patients with the nursing care provided.

Given the distribution of our data and to ensure accurate inter-group comparisons, Fisher exact test was chosen. This decision was made especially relevant due to instances of cell counts <5 in the data matrix, where conventional methods like the chi-square test might not render precise results.

## 3. Results

### 3.1. Patient adherence scores comparison before and after interventions

Before the intervention, there were no statistically significant differences in all the patient adherence behavior scores between the 2 groups (*P* > .05). After the intervention, the adherence behavior scores in both groups improved compared to pre-intervention, demonstrating the effectiveness of the implemented nursing interventions. Specifically, in the experimental group, patients showed higher adherence behavior scores than the control group for aspects such as balanced diet, persistent exercise, regular medication, and regular review visits. These differences were statistically significant (*P* < .05), suggesting that the FTS-based holistic rehabilitation nursing intervention provided substantial improvements in adherence to the prescribed regimen. These results are presented in Table 2.

**Table 2:** Comparison of adherence to medical advice before and after intervention between experimental and control groups (scores, mean ± SD).

Group	Balanced diet	Regular exercise	Timely medication	Regular checkup
Experimental group (pre-intervention)	14.45 ± 1.26	13.45 ± 1.44	14.50 ± 1.37	13.45 ± 1.30
Experimental group (post-intervention)	21.00 ± 1.40	22.20 ± 1.30	21.00 ± 1.44	22.00 ± 1.40
*t*-value	10.450	13.500	10.350	13.850
*P*-value	<.001	<.001	<.001	<.001
Control group (pre-intervention)	14.50 ± 1.40	13.55 ± 1.35	14.50 ± 1.40	13.50 ± 1.30
Control group (post-intervention)	20.40 ± 1.35	21.50 ± 1.40	20.50 ± 1.35	21.50 ± 1.30
*t*-value	7.645	10.625	7.875	10.750
*P*-value	<.001	<.001	<.001	<.001
*t*_1_-value	0.330	0.165	0.135	0.365
*P*_1_-value	.785	.915	.940	.765
*t*_2_-value	2.813	2.730	2.502	2.350
*P*_2_-value	.0095	.0115	.0158	.0295

*Notes*: *t*_1_ and *P*_1_ represent comparison before intervention between 2 groups, *t*_2_ and *P*_2_ represent comparison after intervention between 2 groups.

### 3.2. PSQI comparisons at different time points

Overall, there were statistically significant differences in the PSQI scores between the groups, at different time points, and in the interaction effect (*P* < .05). Upon further pairwise comparisons, within each group, there were statistically significant differences in PSQI scores at different time points (*P* < .05). This indicated the sleep quality changed significantly over time, potentially due to the influence of the nursing interventions. Between the groups, at all the assessed time points, the experimental group had lower PSQI scores compared to the control group. These differences were statistically significant (*P* < .05), suggesting better sleep quality in the experimental group. This improvement can be attributed to the holistic rehabilitation nursing interventions based on the FTS concept, which seemed to contribute to better sleep quality in patients with LEAOD. It is noteworthy to highlight that lower PSQI scores reflect better sleep quality. Hence, the results further underline the positive impact of the FTS-based holistic rehabilitation nursing interventions on both patient adherence and sleep quality. These findings offer insights into how nursing practices can be improved to enhance patient outcomes in LEAOD treatment. These results are presented in Table 3.

**Table 3:** Comparative analysis of PSQI scores in experimental and control groups at different time points.

Group	Number of cases	Post-intervention 3d	Post-intervention 7d	Post-intervention 15d
Experimental group	46	12.95 ± 1.33‡	9.85 ± 1.30*,‡	7.90 ± 1.38*,†,‡
Control group	46	14.95 ± 1.37	11.90 ± 1.42*	8.75 ± 1.59*,†
*F*_Time_, *P*_Time_	435.88, <.001			
F_Group_, *P*_Group_	101.40, <.001			
*F*_Time × Group_, *P*_Time × Group_	5.50, 0.006			

* Compared to this group’s Post-Intervention 3d, *P* < .05.

† Compared to this group’s post-intervention 7d, *P* < .05.

‡ Compared to the control group at the same time, *P* <.05.

### 3.3. Treatment satisfaction outcomes

Upon completion of the interventions, the treatment satisfaction survey revealed notable differences in patients’ contentment between the experimental and control groups.

In the experimental group (n = 46), 15 patients (32.61%) reported being “Satisfied” with the treatment, 29 patients (63.04%) reported “Basic Satisfaction,” and a minimal 2 patients (4.35%) were “Dissatisfied.” This results in an overall satisfaction rate of 95.65% when combining the “Satisfied” and “Basic Satisfaction” responses. Conversely, in the control group (n = 46), 10 patients (21.74%) reported being “Satisfied,” 24 patients (52.17%) indicated “Basic Satisfaction,” and a higher number of 12 patients (26.09%) were “Dissatisfied.” The control group thus had a notably lower overall satisfaction rate of 73.91%.

Using Fisher exact test to assess the significance of the observed differences, a significant disparity in overall satisfaction rates between the 2 groups was observed, with a *P*-value of .007 (<.05). Specifically, the experimental group, which underwent holistic nursing interventions based on the FTS concept, showcased a markedly higher overall satisfaction rate in comparison to the control group. These results are presented in Table 4.

**Table 4:** Comparison of treatment satisfaction rates between experimental and control groups in patients with LEAOD.

Treatment outcome	Experimental group		Control group	
	Number (n = 46)	Rate	Number (46)	Rate
Satisfied	15	32.61%	10	21.74%
Basically satisfied	29	63.04%	24	52.17%
Dissatisfied	2	4.35%	12	26.09%
Total satisfaction	44	95.65%	34	73.91%

*Note*: Fisher exact test *P* = .007 < .05.

## 4. Discussion

The implementation of holistic nursing interventions, under the guidance of the FTS concept, represents an innovative patient-centered approach to rehabilitation planning and care.^[12]^ The research findings from this study underscored the potential of such interventions, with the experimental group exhibiting significantly higher adherence scores than the control group (*P* < .05). LEAOD is a prevalent condition within the field of vascular surgery, characterized by high rates of disability and comparatively poor postoperative prognosis.^[2,13]^ Traditional nursing interventions often rely on rote learning strategies or “force-feeding” of disease-related knowledge, which can lead to a gradual decline in patients’ learning interest and initiative.^[11,14]^ This suggests a need to reconceptualize the way patients are engaged and educated about their health.

Leveraging modern information technologies, healthcare professionals can transform disease-related knowledge into a more engaging and visual format. By promoting an active learning environment, patients can better understand the risks associated with their disease, appreciate the importance of self-care strategies, and become more equipped to independently execute disease-related interventions.^[15,16]^ Such an approach could increase communication between caregivers and patients and improve patient adherence. During surgery, effective pressure reduction in the operation area facilitates peripheral blood flow, prevents skin pressure injuries, and avoids hypothermia due to massive fluid loss. These actions lay a solid foundation for the patient’s early recovery postoperatively.

Moreover, our repeated measures variance analysis of the post-intervention sleep quality at 3, 7, and 15 days revealed that, as the nursing intervention time continued, the PSQI scores of the experimental group were consistently lower than those of the control group (*P* < .05). This finding indicates that guiding patients to engage in early postoperative rehabilitative exercise increases peripheral capillary density and stimulates the revitalization and regeneration of surrounding muscles and nerve cells at the ankle joint.^[17]^ This intervention may accelerate peripheral lymphatic return, prevent limb edema, avoid skin flap necrosis, and enhance ankle joint mobility. Local cold compresses can stimulate surface skin and blood vessels, accelerating blood coagulation, reducing vascular permeability, alleviating tissue edema around the wound, and reducing peripheral nerve sensitivity. These strategies divert patients’ attention from wound pain and coupled with guided relaxation and positive visualization, can lower neuronal excitability and boost endorphin release. Such a therapeutic approach can soothe postoperative patients in a state of high tension, improve sleep quality, and accelerate early recovery.^[18,19]^

In conclusion, for patients with LEAOD, holistic nursing interventions guided by the FTS concept can lead to increased adherence, improved sleep status, and contribute significantly to the sustainable development of healthcare institutions. By shifting the paradigm of patient care from a passive to a more active and engaging learning environment, we can empower patients to play a more proactive role in their treatment and recovery process. Furthermore, these interventions can also improve the quality of perioperative care, thereby enhancing postoperative outcomes and accelerating early recovery. Therefore, this study offers important insights into how the principles of FTS can be integrated into nursing practice, with the potential to significantly improve patient outcomes in the context of LEAOD management. The results also underscore the need for further research to explore the application and impacts of such holistic nursing interventions in other contexts and patient populations.

Despite the valuable insights provided by our study into the application of holistic nursing interventions based on the FTS concept for patients with lower extremity arterial occlusive disease, it is crucial to acknowledge its limitations. Our study was limited by a relatively small sample size, which may restrict the statistical power and external validity of our results. This was compounded by the fact that the research was carried out in a single medical institution, possibly confining the diversity of patient data and healthcare practices examined. Furthermore, the focus was primarily on the immediate postoperative period up to 15 days, thereby neglecting potential long-term impacts of the interventions. The use of self-reported measures for assessing outcomes such as sleep quality and patient adherence could also be subject to recall bias or social desirability bias. Finally, the absence of randomization or blinding procedures might introduce potential biases. Additionally, the study did not evaluate patients’ psychological well-being or exercise tolerance, which could offer a more comprehensive understanding of the intervention’s impact. Although these limitations should be considered when interpreting our results, they also offer a roadmap for future research to enhance these areas, leading to more robust and universally applicable findings.

## 5. Conclusions

In conclusion, our study underscores the potential benefits of integrating holistic nursing interventions based on the FTS concept for patients with lower extremity arterial occlusive disease. These interventions significantly improved patient adherence and sleep quality, thereby potentially enhancing postoperative recovery and overall health outcomes. Despite the inherent limitations of our study, the findings provide a solid foundation for future research to explore the full potential of holistic nursing approaches in the context of FTS.

## Acknowledgments

We appreciate the cooperation and informed consent provided by the patients for this study.

## Author contributions

**Data curation:** Jie Zhang.

**Formal analysis:** Jing Huang.

**Investigation:** Guojun Zeng.

**Methodology:** Lin Zhang.

**Writing – original draft:** Jie Zhang.

**Writing – review & editing:** Xiaoyan Liu.
